# Intrafraction Motion and Margin Assessment for Ethos Online Adaptive Radiotherapy Treatments of the Prostate and Seminal Vesicles

**DOI:** 10.1016/j.adro.2023.101405

**Published:** 2023-11-04

**Authors:** Mikel Byrne, Amy Yuen Meei Teh, Ben Archibald-Heeren, Yunfei Hu, James Rijken, Suhuai Luo, Trent Aland, Peter Greer

**Affiliations:** a Icon Cancer Centre, South Brisbane, Queensland, Australia; b School of Information and Physical Sciences, University of Newcastle, Newcastle, New South Wales, Australia; c Sydney Adventist Hospital, Wahroonga, New South Wales, Australia; d ANU College of Health and Medicine, The Australian National University, Canberra, Australian Capital Territory, Australia; e Calvary Mater Newcastle Hospital, Newcastle, New South Wales, Australia

## Abstract

**Purpose:**

Online adaptive radiation therapy (OART) uses daily imaging to identify changes in the patient's anatomy and generate a new treatment plan adapted to these changes for each fraction. The aim of this study was to determine the intrafraction motion and planning target volume (PTV) margins required for an OART workflow on the Varian Ethos system.

**Methods and Materials:**

Sixty-five fractions from 13 previously treated OART patients were analyzed for this retrospective study. The prostate and seminal vesicles were contoured by a radiation oncologist on 2 cone beam computed tomography scans (CBCT) for each fraction, the initial CBCT at the start of the treatment session, and the verification CBCT immediately before beam-on. In part 1 of the study, PTVs of different sizes were defined on the initial CBCT, and the geometric overlap with the clinical target volume (CTV) on the verification CBCT was used to determine the optimal OART margin. This was performed with and without a patient realignment shift by registering the verification CBCT to the initial CBCT. In part 2 of the study, the margins determined in part 1 were used for simulated Ethos OART treatments on all 65 fractions. The resultant coverage to the CTV on the verification CBCT, was compared with an image guided radiation therapy (IGRT) workflow with 7-mm margins.

**Results:**

Part 1 of the study found, if a verification CBCT and shift is performed, a 4-mm margin on the prostate and 5 mm on the seminal vesicles resulted in 95% of the CTV covered by the PTV in >90% of fractions, and 98% of the CTV covered by the PTV in >80% of fractions. Part 2 of the study found when these margins were used in an Ethos OART workflow, they resulted in CTV coverage that was superior to an IGRT workflow with 7-mm margins.

**Conclusions:**

A 4mm prostate margin and 5-mm seminal vesicles margin in an OART workflow with verification imaging are adequate to ensure coverage on the Varian Ethos system. Larger margins may be required if using an OART workflow without verification imaging.

## Introduction

When planning radiation therapy, a clinical target volume (CTV) is defined which indicates the gross tumor volume and any subclinical disease.^1^ A margin is added to the CTV to create the planning target volume (PTV),^1^ which is used to ensure the CTV receives the prescribed dose within an acceptable range considering process uncertainties. The most commonly used method of calculating the margin required was proposed by Van Herk et al,^2^^,^^3^ where to ensure a minimum dose to the CTV of 95% of the prescribed dose for 90% of patients, the margin required is 2.5 Σ + 0.7 σ, where Σ represents the standard deviation of systematic errors, and σ represents the standard deviation of random errors.

Prostate adenocarcinoma is commonly and successfully treated with radiation therapy, with 60 Gy in 20 fractions a commonly used dose regimen.^4^ The margin used in prostate radiation therapy with an image guided radiation therapy (IGRT) workflow is typically 5 to 8 mm,^5^ with 7 mm a common value depending on a range of factors in the treatment workflow.^6^^,^^7^

Online adaptive radiation therapy (OART) is a process where the patient's anatomy is imaged for each fraction, and a new treatment plan is generated based on the position of the anatomy in a given fraction.^8^ OART changes many of the uncertainties that occur in the treatment workflow and therefore necessitates a re-evaluation of the margins required.^9^ For example, interfraction motion no longer needs to be accounted for as a new plan is generated each day, whereas intrafraction motion may increase due to the longer treatment times, and contouring uncertainty may be considered a random rather than systematic error necessitating a much smaller margin. OART has the potential to result in both greater target coverage and less dose to organs at risk (OARs) both with^10^, ^11^, ^12^, ^13^, ^14^, ^15^, ^16^, ^17^, ^18^ and without^19^^,^^20^ margin reductions, although the largest benefits reported with OART come from margin reductions.^10^, ^11^, ^12^, ^13^, ^14^, ^15^, ^16^, ^17^

OART has recently become clinically feasible with the introduction of systems such as the Ethos system (Varian Medical Systems). The Ethos OART workflow requires a cone beam computed tomography scan (CBCT) to be acquired for online adaptive planning and allows the user the option to acquire a verification CBCT after online adaptive planning is complete before delivering the treatment. The treatment couch position can be adjusted based on the verification image to compensate for intrafraction motion. Full details of the Ethos OART system are discussed by Archambault et al.^21^

Several recent studies investigate margins for prostate OART,^22^, ^23^, ^24^, ^25^ but do not consider all the uncertainties specific to the Ethos OART workflow,^22^^,^^24^ or apply to slightly different anatomic situations (postprostatectomy without seminal vesicles).^23^^,^^25^ This study aims to investigate the intrafraction motion and margins required for OART with the Ethos system to the prostate and seminal vesicles. The study is broken into 2 parts; part 1 aims to use a simplified geometric model to determine the optimal margin for OART, and part 2 aims to verify that when this margin is used for OART in the Ethos system, including all process uncertainties, target coverage is not compromised compared with IGRT. For parts 1 and 2 of the study, the effect of omitting a verification image from the adaptive workflow was also assessed.

## Methods and Materials

Sixty-five fractions from 13 patients who were previously treated with OART on the Ethos v1.1 system were selected for this retrospective study. The patients previously received radiation therapy to the prostate and seminal vesicles, and in some cases also the regional lymph nodes, although these were ignored for the purposes of this study. All patients gave informed consent for their data to be used in this research and research was conducted in accordance with the Code of Ethics of the World Medical Association (Declaration of Helsinki) for experiments involving humans. The research was categorized as negligible ethical risk and exempt from ethics committee review.

In preparation for simulation and treatment, per department standard practice, patients were instructed to stay well hydrated in the hours leading up to treatment and to have their final drink of 400 mL of water 1 to 2 hours before treatment. Patients were then advised to have their final attempt at emptying their bladder and rectum 45 to 60 minutes before treatment. This guidance was adjusted according to patient tolerance with the aim of achieving a comfortably filled bladder, which had reached steady-state by the time of treatment to minimize intrafractional bladder filling. The patient was also recommended a diet to minimize gas within the gastrointestinal tract.

During OART, per standard departmental practice, patients received 2 CBCT images per fraction: a pretreatment image used for online adaptive replanning (CBCT_init_), and a verification image acquired immediately before beam-on to correct for any intrafraction motion that occurred during the planning stage of the adaptive treatment (CBCT_verif_). The same imaging parameters were used for the 2 CBCTs.

For this study, CBCT images acquired from fractions 1, 5, 9, 13, and 17 were exported from Ethos and imported into Eclipse v16.1 (Varian Medical Systems) for analysis for each patient chosen to span the treatment course. Two different rigid registrations were created between the CBCT_init_ and CBCT_verif_ images for each fraction, namely the soft tissue– based online registration that was manually performed during the treatment fraction and applied to the treatment couch (REG_verif_ representing the transformation matrix for the soft tissue based registration), and a registration to the DICOM isocenter for the scenario where no verification image was acquired and no couch shift applied (REG_No verif_ representing the transformation matrix for the DICOM isocenter-based registration).

A radiation oncologist (RO) then contoured the prostate and seminal vesicles de novo on the CBCT_init_ and CBCT_verif_ of each fraction of each patient. All structures were set to the highest resolution setting available in the Eclipse system. For the purposes of this study the prostate and seminal vesicles were considered the CTVs. All patient data were de-identified.

### Fraction time

As intrafraction motion is known to increase as the fraction time increases,^26^, ^27^, ^28^ the time between acquiring the CBCT_init_ and CBCT_verif_ is reported to assist with interpretation of margin results. Timing information was extracted from the image DICOM headers.

## Part 1: Intrafraction Motion/PTV Margin Assessment

**Figure 1:**
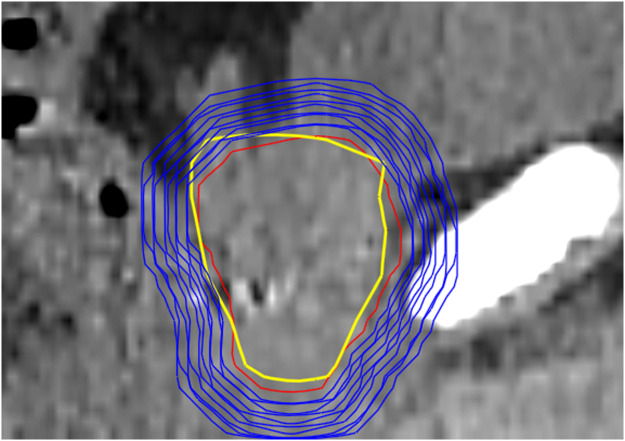
Sagittal visualization of initial cone beam computed tomography prostate clinical target volume (red), different planning target volume margins (blue), and verification cone beam computed tomography prostate clinical target volume (yellow) for an example cone beam computed tomography.

To assess the intrafraction motion, PTVs were created in 1-mm isotropic increments up to 10 mm and added to the prostate and seminal vesicles as contoured on CBCT_init_ (Fig. 1). CBCT_init_ was registered to CBCT_verif_ using REG_verif_, and the overlap of the PTVs on CBCT_init_ with the prostate and seminal vesicles on CBCT_verif_ was checked, to represent an adaptive workflow with verification imaging. The process was then repeated but this time applying REG_no verif_, to represent an adaptive workflow without verification imaging. The percentage of fractions that the CTV defined on the CBCT_verif_ was covered by different PTV margins were then determined for both workflows, as well as the motion of the structure center of mass (COM). The smallest margin in the verification shift (REG_verif_) workflow that met the 2 following criteria was determined as the OART optimized margin:

1) 95% of the CTV is covered by the PTV in 90% of fractions.
2) 98% of the CTV is covered by the PTV in 80% of fractions.

## Part 2: Online Adaptive Dosimetric Coverage Assessment

The results from part 1 of this study allow determination of the overlap of the PTV (created from the initial position of the CTV) with the CTV at the time of beam-on in an online adaptive context. However, in practice the PTV is not always fully covered by the prescribed dose, and therefore overlap with the PTV does not always guarantee coverage of the CTV. In addition, in the Ethos adaptive workflow the prostate and seminal vesicles are created by the artificial intelligence (AI) algorithms and need to be adjusted by the treatment team to create the PTV on a given day. Therefore, the margins determined in part 1 were used to calculate the resultant coverage when all uncertainties in the Ethos treatment process are taken into account.

Part 2 of the study evaluates the CTV coverage achievable with 3 different potential treatment workflows. These are

1) An IGRT workflow with margins of 7 mm.
2) An adaptive workflow with reduced margins (as determined from part 1), with a verification image acquired and shifts applied immediately before beam-on.
3) An adaptive workflow with reduced margins (as determined from part 1), without a verification image before beam-on (used at some institutions to reduce additional imaging).

**Figure 2:**
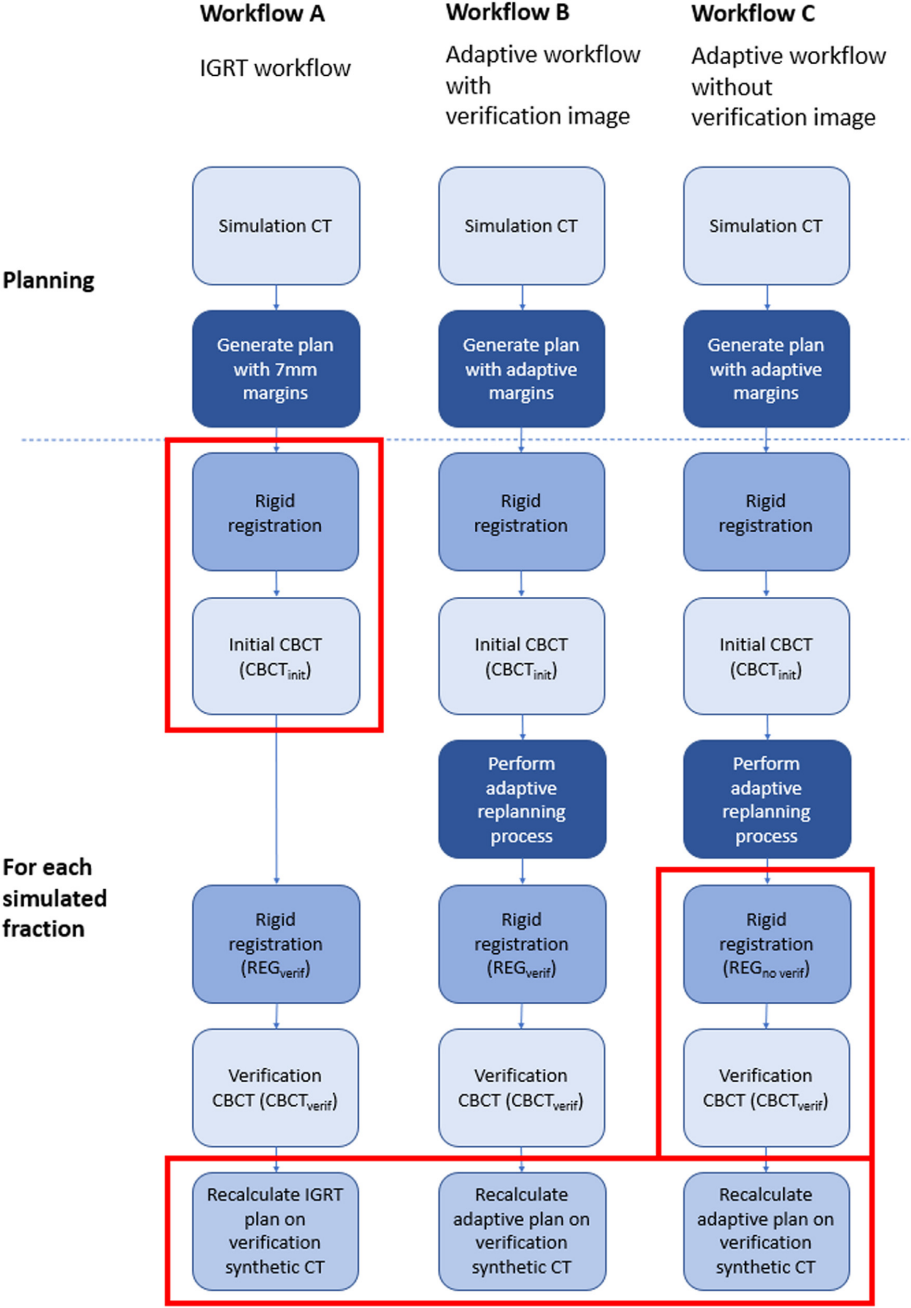
Diagram illustrating the steps used to simulate each workflow in the study. All steps shown were performed in this study; the red boxes highlight steps that would not normally occur as part of the clinical workflow that was being simulated. These highlighted steps were performed in this study to facilitate the data extraction and analysis. Note the only difference between workflow A and workflow B is the margins used for planning and the adaptive replanning process. Note the only difference between workflow B and workflow C is the registration used for the CBCT_verif_. *Abbreviations*: CBCT = cone beam computed tomography; CT = computed tomography; IGRT = image guided radiation therapy.

A diagrammatic representation of the study process used for workflows A, B, and C is shown in Fig. 2. Plans were generated for each workflow with a prescription of 60 Gy/20 fractions in the Ethos v1.1 treatment planning system, setting the rectum, bladder, prostate, and seminal vesicles as “influencer” structures. The CTVs were set as independent structures and were not derived from the influencer structures, whereas PTVs were derived from CTVs. Simulated treatments were carried out by staff that had passed in-house Ethos credentialling tests and were experienced in delivering clinical OART treatments. Staff were instructed to perform simulated treatments following clinical workflows but were not under time pressure. They adjusted the system generated structures as needed until satisfied the targets and OARs were suitable for adaptive planning, with the expectation of greater than 2-mm accuracy in areas within 3 cm of the targets. The synthetic CTs used for dose calculation were generated in the Ethos workflow using deformable image registration (mutual information image similarity and b-spline regularization) of the planning CT.

### Workflow A

The patients were planned on the simulation CT with 7-mm isotropic margins to the prostate and seminal vesicles, which was created to represent a typical IGRT approach. The planning CT was rigidly registered to the CBCT_init_ using the Ethos OART scheduled plan matching algorithm,^29^ and then registered to CBCT_verif_ using REG_verif_. This effectively meant that the IGRT plan registration was carried out to the CBCT_verif_ image acquired immediately before treatment delivery, replicating a standard IGRT workflow. The IGRT plan was then recalculated on the synthetic CT generated from the CBCT_verif_ (to represent dose resulting from workflow A).

### Workflow B

An online adaptive plan using identical settings but with the reduced margins determined in part 1 above was created. Simulated adaptive replanning in the Ethos system was then carried out on the CBCT_init_. In this process, online adaptive plans were generated based on AI- and user-defined CTVs. The CBCT_init_ was then registered to CBCT_verif_ using the REG_verif_ and the online adaptive plan recalculated on the synthetic CT generated from CBCT_verif_ (to represent dose resulting from workflow B).

### Workflow C

Online adaptive plans were calculated as per workflow B. The CBCT_init_ was then registered to CBCT_verif_ using REG_no verif_ to represent no verification imaging shift, and the online adaptive plan recalculated on the synthetic CT generated from CBCT_verif_ (to represent dose resulting from workflow C).

Results are presented using a range of target coverage metrics to the oncologist defined prostate and seminal vesicle CTVs on CBCT_verif_, and dose to the whole-body structure. For each metric the Hodges-Lehmann estimate of the median is shown, as well as the percentage of fractions in which ideal values are met for a subset of the metrics.

Estimation of the effect of workflow on each radiation therapy metric was performed using mixed effects ordinal logistic regression. Within each model, fixed effects of workflow and fraction number were included and a random effect for patient was included to account for the nonindependence of measurements from the same patient. Results presented include odds ratios (ORs) and contrast *P* values. The presented ORs correspond to the odds of achieving a higher metric value between workflows and are reported with 95% CIs.

All statistical analyses were programmed using SAS v.9.4 (SAS Institute Inc). The null hypothesis (H_0_) was that there was no difference between the IGRT plan and the adaptive plan for the patient group. Statistical significance was set a priori at *P* < .05.

## Results

### Fraction time

The average time between acquiring the CBCT_init_ and CBCT_verif_ was 17.1 minutes, with a standard deviation of 5.8 minutes. Both average fraction delivery time and the variation in fraction time decreased as the course progressed (average of 18.6 ± 8.6 minutes for fraction 1, and 15.5 ± 4.9 minutes for fraction 17).

## Part 1: Intrafraction Motion

**Figure 3:**
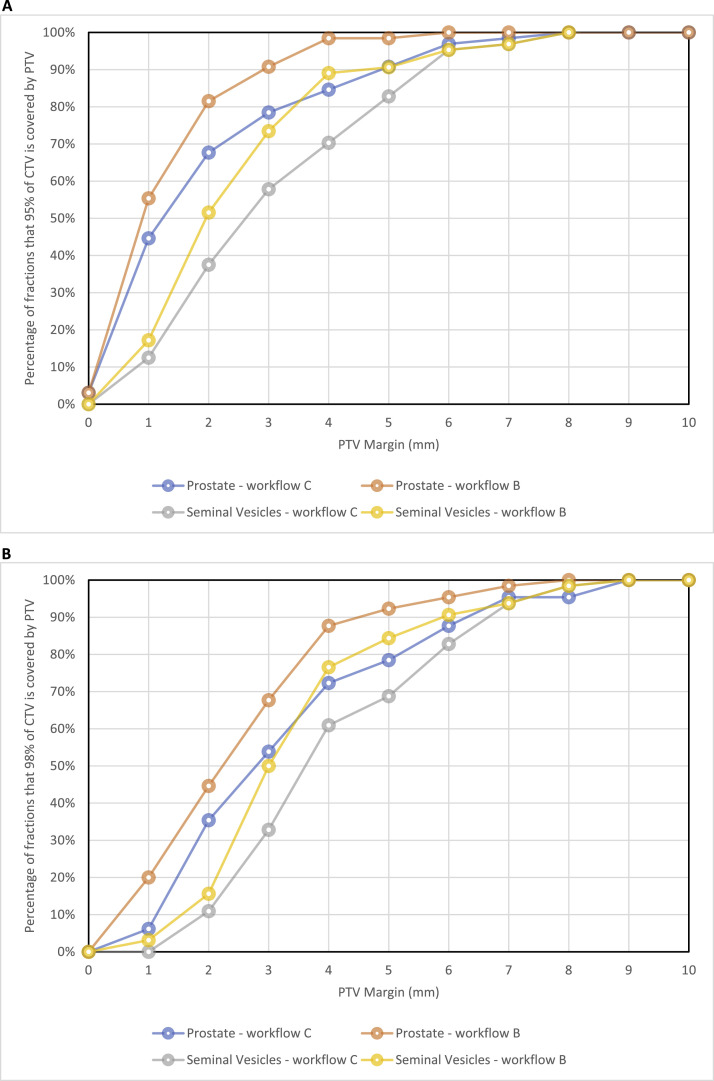
(A) Graph of the percentage of fractions that the PTV would cover 95% of the CTV for a range of different margins. (B) Graph of the percentage of fractions that the PTV would cover 98% of the CTV for a range of different margins. Note: Workflow B = with verification imaging shift applied; Workflow C = no verification imaging shift. *Abbreviations:* CTV = clinical target volume; PTV = planning target volume.

The percentage of fractions that had 95% and 98% of the volume of the CTV covered by the PTV with different margin expansions is shown in Fig. 3a and b, respectively. The smallest margins for the prostate and seminal vesicle PTVs ensuring 95% CTV coverage in 90% of fractions were 5 and 6 mm without verification imaging, and 3 and 5 mm with verification imaging, respectively. The smallest PTV margins ensuring 98% CTV coverage in 80% of fractions were 6 mm for both prostate and seminal vesicles without verification imaging, and 4 mm for the prostate and 5 mm for seminal vesicles with verification imaging. Considering only a workflow that uses verification imaging, the smallest margin that meets both criteria is 4 mm for the prostate and 5 mm for the seminal vesicles.

Table 1 is included to allow comparison with other studies that use COM of the structures or fiducials. The average COM shift was <1 mm in all directions, except for workflow C (ie, without a verification image shift) where the average shift exceeded 1 mm in the patient posterior direction. This indicates on average the structures moved posteriorly during the fraction.

**Table 1:** CTV volume and magnitude of motion calculated from the center of mass of the structures both with verification imaging shift (workflow B) and without (workflow C)

		CTV volume (cm^3^)		COM shift (mm) (average ± SD)			
Structure	Workflow	CBCT_init_	CBCT_verif_	Lateral	Vertical	Longitudinal	3D motion
Prostate	Workflow B	57.1 ± 26.1	58.2 ± 27.2	0.0 ± 0.7	0.3 ± 1.6	–0.2 ± 1.8	2.2 ± 1.3
	Workflow C			0.3 ± 1.3	1.2 ± 2.4	–0.7 ± 2.5	3.3 ± 2.2
Seminal vesicles	Workflow B	14.9 ± 7.8	15.7 ± 8.8	0.0 ± 1.5	0.4 ± 1.9	0.1 ± 2.1	2.8 ± 1.5
	Workflow C			0.3 ± 1.8	1.5 ± 2.6	0.0 ± 2.5	3.7 ± 2.0

*Abbreviations:* 3D = 3-dimensional; CBCT = cone beam computed tomography; COM = center of mass; CTV = clinical target volume.

Positive lateral, vertical, and longitudinal values indicate a shift to the patient's left, posterior, and superior, respectively during the fraction. Note that individual components are averaged as vectors, whereas 3D motion is calculated as a scalar quantity (average of the square root of the sum of squares of each component).

## Part 2: Online Adaptive Coverage Assessment

The margin values determined in part 1 were used to perform simulated treatments for part 2 of the study. The results of part 2 of the study are shown in Table 2. The ORs presented compare the odds of achieving a given dose or volume metric between treatment workflows, with ORs >1 indicating increased odds of a higher metric between 2 workflows and ORs <1 indicating decreased odds of achieving a higher metric value between 2 workflows. For example, a comparison of workflow B versus A indicated that patients were 20.30 times as likely to have a higher prostate D0.35 cc metric with workflow B (OR, 20.30; 95% CI, 9.42-43.78) and this was found to be statistically significant (*P* < .001).

**Table 2:** Hodges-Lehmann median and statistical testing for the plan metrics analyzed

	Workflow A: IGRT	Workflow B: Adaptive with verification image				Workflow C: Adaptive without verification image				
Structure and metric	Hodges-Lehmann median	Hodges-Lehmann median	Odds ratio (B vs A)	Contrast *P* value	Reject (R) or fail to reject (FTR) H_0_	Hodges-Lehmann median	Odds ratio (C vs A)	Contrast *P* value	Reject (R) or fail to reject (FTR) H_0_	Superior workflow
Prostate D0.35 cc	62.8 Gy	63.3 Gy	20.3 (9.42, 43.78)	<.001	R	63.4 Gy	27.73 (12.63, 60.89)	<.001	R	A
Prostate D80%	61.1 Gy	61.8 Gy	29.14 (13.27, 63.98)	<.001	R	61.8 Gy	28.19 (12.68, 62.66)	<.001	R	B
Prostate D90%	60.7 Gy	61.6 Gy	33.21 (15.10, 73.05)	<.001	R	61.5 Gy	24.38 (11.09, 53.55)	<.001	R	B
Prostate D95%	60.2 Gy	61.3 Gy	14.56 (7.03, 30.17)	<.001	R	61.0 Gy	7.42 (3.65, 15.10)	<.001	R	B
Prostate D98%	59.6 Gy	60.7 Gy	5.4 (2.76, 10.57)	<.001	R	60.0 Gy	2.35 (1.21, 4.57)	.014	R	B
Prostate V54 Gy	100.0%	100.0%	0.09 (0.03, 0.29)	<.001	R	99.9%	0.04 (0.01, 0.15)	<.001	R	A
Prostate V57 Gy	100.0%	99.8%	0.31 (0.14, 0.66)	.004	R	99.6%	0.16 (0.07, 0.34)	<.001	R	A
Prostate V60 Gy	96.0%	98.9%	7.88 (3.95, 15.76)	<.001	R	98.0%	3.88 (1.98, 7.59)	<.001	R	B
Seminal vesicles D0.35 cc	62.6 Gy	63.1 Gy	13.5 (6.42, 28.35)	<.001	R	63.1 Gy	12.59 (6.05, 26.22)	<.001	R	A
Seminal vesicles D80%	60.7 Gy	61.7 Gy	19.46 (9.09, 41.68)	<.001	R	61.6 Gy	13.54 (6.39, 28.66)	<.001	R	B
Seminal vesicles D90%	60.4 Gy	61.3 Gy	13.35 (6.49, 27.44)	<.001	R	61.2 Gy	8.72 (4.27, 17.82)	<.001	R	B
Seminal vesicles D95%	60.0 Gy	61.0 Gy	8.27 (4.15, 16.47)	<.001	R	60.8 Gy	5.02 (2.55, 9.86)	<.001	R	B
Seminal vesicles D98%	59.6 Gy	60.4 Gy	4.7 (2.42, 9.13)	<.001	R	60.0 Gy	2.46 (1.30, 4.69)	.008	R	B
Seminal vesicles V54 Gy	100.0%	100.0%	0.99 (0.41, 2.37)	.975	FTR	100.0%	0.64 (0.28, 1.48)	.282	FTR	A
Seminal vesicles V57 Gy	99.9%	99.9%	0.9 (0.41, 1.96)	.784	FTR	99.7%	0.6 (0.28, 1.28)	.178	FTR	A
Seminal vesicles V60 Gy	92.0%	98.7%	4.4 (2.23, 8.69)	<.001	R	97.9%	2.38 (1.23, 4.60)	.012	R	B
Body V30 Gy	811.7 cc	596.9 cc	0.01 (0.00, 0.02)	<.001	R	597.2 cc	0.01 (0.00, 0.02)	<.001	R	B
Body V57 Gy	202.1 cc	158.9 cc	0.01 (0.00, 0.01)	<.001	R	159.1 cc	0.01 (0.00, 0.01)	<.001	R	B
Body V60 Gy	149.8 cc	129.6 cc	0.04 (0.02, 0.10)	<.001	R	129.9 cc	0.04 (0.02, 0.10)	<.001	R	B

*Abbreviation*: IGRT = image guided radiotherapy.

The superior workflow reported is that which gave the lowest dose for body and D0.35 cc metrics, and that which gave the highest dose for other target coverage–related metrics. The null hypothesis (H_0_) was that there is no difference in plan metric between the IGRT plan and the adaptive plan.

Considering the verification image workflow (workflow B), of the 14 metrics in Table 2 that represent target coverage, 10 showed significant improvements using workflow B, 2 showed significant improvements for workflow A and 2 were not statistically significant. Similarly considering the nonverification imaging workflow (workflow C), 10 showed significant improvements with workflow C, 2 showed improvements with workflow A, and 2 were not statistically significant.

In Table 3, the results above are presented in terms of percentage of fractions that a given coverage goal was met, which is more akin to how margins are typically calculated. Workflow A met the 54 Gy and 57 Gy prostate CTV goals more frequently than other workflows. Workflow B met all other goals more frequently than the other workflows.

**Table 3:** Percentage of fractions that different goals related to target coverage were met for each workflow

		Percentage of fractions where goal is met		
Structure	Goal	Workflow A: IGRT	Workflow B: Adaptive with verification	Workflow C: Adaptive without verification
Prostate CTV	D95 > 90% (54 Gy)	100.0%	98.5%	95.4%
	D95 > 95% (57 Gy)	100.0%	98.5%	93.8%
	D95 > 100% (60 Gy)	63.1%	89.2%	81.5%
Seminal vesicles CTV	D95 > 90% (54 Gy)	93.8%	96.9%	96.9%
	D95 > 95% (57 Gy)	89.2%	96.9%	93.8%
	D95 > 100% (60 Gy)	52.3%	89.2%	80.0%

*Abbreviations*: CTV = clinical target volume; IGRT = image guided radiotherapy.

## Discussion

The results from part 1 indicate the intrafraction prostate motion seen in this cohort of patients is marginally larger than has been reported in some other studies.^23^^,^^25^^,^^26^^,^^30^, ^31^, ^32^ Our results suggest that prostate margins of 4 mm are required with OART. Other studies have reported intrafraction margins of 2 to 5 mm,^23^, ^24^, ^25^, ^26^, ^27^^,^^30^, ^31^, ^32^, ^33^ depending on a range of factors in the treatment workflow. Morgan et al^23^^,^^25^ found a 3-mm margin was sufficient for OART treatment of the prostatic fossa over an average treatment time of 10.7 minutes. Using a magnetic resonance (MR)-guided OART workflow with an average treatment time of 33.1 minutes de Muinck Keizer et al^24^ found a 5-mm margin was adequate to maintain coverage.

The results from part 1 suggest a 5-mm margin is required for coverage of the seminal vesicles. This result matched the findings of Sheng et al^22^ during a period of “20 to 30 minutes,” and is consistent with known intrafraction motion of the seminal vesicles.^34^

We have used the methodology of Sheng et al,^22^ which has the advantage that it accounts for deformation and relative motion between the prostate and seminal vesicles; however, only considers isotropic margin expansions. Further reduction may be possible if using anisotropic margins. Other potential contributors to the difference in findings between our study and other reported studies may also include

1) Other studies have reported shorter treatment periods than observed in this study.^26^^,^^30^ A study by Li et al^30^ suggested 2-mm margins are adequate over an average treatment time of 8.7 minutes. As time increases, prostate displacement is known to increase.^26^, ^27^, ^28^^,^^35^ Our study observed average time, measured as the time between CBCT_init_ and CBCT_verif_, to be 17.1 ± 5.8 minutes.
2) It is possible that adjustments can be made to patient bladder and rectal preparation to further minimize intrafractional motion of the bladder and rectum. A bladder filling protocol that minimizes filling during treatment is generally preferred for OART.^36^
3) Many intrafraction motion studies use fiducials or COM to assess motion of the prostate.^25^^,^^26^^,^^30^ These studies often consider the prostate as a rigid structure that only translates and rotates, and do not account for deformation of the prostate over the course of the fraction. Like Sheng et al^22^ we found that the COM (Table 1) underestimates the margin required. By contouring the full prostate and seminal vesicles the method used in this study accounts for prostate and seminal vesicle deformation, but also includes contouring uncertainty. Contouring uncertainty can be substantial,^37^ although interobserver variations have been reduced by having a single oncologist performing all the contouring for each patient in part 1 of the study. Even so, it is likely the uncertainty in contouring, particularly at the prostate apex, which is difficult to visualize on CBCT, has contributed to the slightly larger margins seen here. It is difficult to separate the effects of deformation and contouring uncertainty; however, both can occur in the clinical workflow and were included in part 2 of this study.

It is important that dosimetric target coverage and outcomes resulting from margin reductions are equivalent to previous methods, as indicated by the studies of Engels et al.^38^^,^^39^ When the reduced margins determined in part 1 were put into the Ethos workflow with verification imaging for part 2 of the study, coverage was maintained. The statistically significant results related to target coverage favored the adaptive plan, indicating that coverage improved using reduced margins in combination with an adaptive workflow. The body structure also shows the patient volume receiving 30 Gy is reduced by 26%, and the volume receiving 57 Gy is reduced by 21%. Due to the amount of data already presented we have decided not to present results for OARs in this study; however, given the reduction in dose to the body it stands to reason that OAR doses should also reduce considerably, as has been found by several other authors.^10^, ^11^, ^12^, ^13^, ^14^, ^15^, ^16^, ^17^

When these reduced margins were used in an Ethos workflow without verification imaging, the results were similar but marginally inferior. As seen in Table 3, both adaptive planning methods have more than 95% of the CTV volume covered by 95% of the prescribed dose in more than 90% of fractions, which is similar to the criteria specified by Van Herk^2^^,^^3^ that the CTV receives ≥95% coverage for 90% of the population. The IGRT plan failed to meet this criterion for the seminal vesicles.

In Table 3 it can also be noted that the IGRT plan tends to have better coverage at the lower isodoses, but lower coverage at the higher isodoses. The planning goals used (for both IGRT and adaptive) specified coverage of the prescription dose to the CTV, and a minimum of 95% of the prescription to the PTV. This tended to lead to plans where the prescription isodose (60 Gy) tightly conformed to the CTV and the 95% isodose (57 Gy) conformed to the PTV. The prescription isodose therefore had almost no margin, such that when any motion occurred it no longer covered the CTV. This was most pronounced for the IGRT plan as it incorporated both inter- and intrafraction motion. In contrast, as the 95% isodose conformed to the PTV, it was much larger in the IGRT case, allowing for maintenance of coverage with the 95% isodose even when motion occurred. There may be scope for future studies to adjust the goals to improve coverage in an OART context.

In the adaptive cases, in the small number of fractions where the intrafraction motion exceeded the margin applied (expected to be <10% of fractions from part 1 of the study), the drop in coverage to the CTV was more pronounced than seen in the IGRT cases.

This study assumes that the dose as calculated is what is delivered. This is not strictly true; however, it has previously been shown the Ethos and Eclipse treatment planning systems perform accurately within acceptable tolerances,^40^ and the geometric uncertainty of the Ethos/Halcyon system^41^ is small compared with intrafraction motion seen here, such that when combined in quadrature with other uncertainties it is unlikely to affect the results of this study.

As the patient's treatment course progressed it was found that treatment time and associated intrafraction motion decreased. One possible explanation for this may be greater patient compliance with bowel and bladder preparation instructions as the patient becomes more familiar with the process. Another potential cause is that the treatment team tends to become familiar with the case and completes the treatment more quickly.

**Figure 4:**
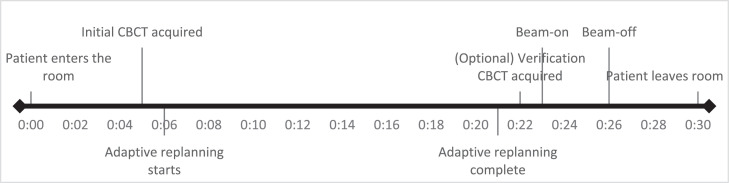
Indicative patient timeline showing when images were acquired relative to treatment delivery (h:mm). *Abbreviation*: CBCT = cone beam computed tomography.

The images used in this study were the preadaptive image taken after setting up the patient, and the pretreatment image taken immediately before beam-on. This does not encompass intrafraction motion that occurs during beam-on and therefore does not encompass the full intrafraction motion that occurs. However, the beam-on time was approximately 3 minutes, which is much shorter than the adaptive plan generation time, which was 17 minutes on average, and therefore we would argue that the bulk of intrafractional motion has been captured (the indicative patient timeline is shown in Fig. 4). Moreover, provided future treatments including beam-on are able to be completed within 17 minutes, as appears to be possible,^20^ the results of this study would be applicable and representative of the intrafraction motion that occurs.

## Conclusion

The results of this study indicate that intrafraction motion can be considerable during the longer fraction times that occur in an Ethos online adaptive workflow. We found that the use of an OART workflow, including verification imaging, allowed margins to be reduced from 7 mm down to 4 mm for the prostate and 5 mm for the seminal vesicles without compromising coverage. If an OART workflow is performed without verification imaging, larger margins may be required. Reduction in margins of this magnitude leads to significant reductions in dose to the patient's normal tissue.

## Disclosures

Mikel Byrne, Amy Yuen Meei Teh, and Ben Archibald-Heeren have received honoraria for presenting on behalf of Varian Medical Systems. The research was partially supported by a research grant from Varian Medical Systems, the manufacturer of Ethos.

## References

[1] Hodapp N (2012). The ICRU Report 83: Prescribing, recording and reporting photon-beam intensity-modulated radiation therapy (IMRT). Strahlenther Onkol.

[2] van Herk M (2004). Errors and margins in radiotherapy. Semin Radiat Oncol.

[3] van Herk M, Remeijer P, Rasch C, Lebesque JV (2000). The probability of correct target dosage: dose-population histograms for deriving treatment margins in radiotherapy. Int J Radiat Oncol Biol Phys.

[4] Dearnaley D, Syndikus I, Mossop H (2016). Conventional versus hypofractionated high-dose intensity-modulated radiotherapy for prostate cancer: 5-year outcomes of the randomised, non-inferiority, phase 3 CHHiP trial. Lancet Oncol.

[5] EviQ Cancer Treat. Cancer Institute NSW 3370-Prostate adenocarcinoma definitive EBRT hypofractionation. Accessed April 8, 2021.https://www.eviq.org.au/radiation-oncology/urogenital/prostate/3370-prostate-adenocarcinoma-definitive-ebrt-hypof

[6] Martin JM, Supiot S, Keall PJ, Catton CN (2018). Moderately hypofractionated prostate external-beam radiotherapy: An emerging standard. Br J Radiol.

[7] Yartsev S, Bauman G (2016). Target margins in radiotherapy of prostate cancer. Br J Radiol.

[8] Lim-Reinders S, Keller BM, Al-Ward S (2017). Online adaptive radiation therapy. Int J Radiat Oncol Biol Phys.

[9] van der Bijl E, Remeijer P, Sonke J-J, van der Heide UA, Janssen T (2022). Adaptive margins for online adaptive radiotherapy. Phys Med Biol.

[10] Grønborg C, Vestergaard A, Høyer M (2015). Intra-fractional bladder motion and margins in adaptive radiotherapy for urinary bladder cancer. Acta Oncol.

[11] Kron T, Wong J, Rolfo A, Pham D, Cramb J, Foroudi F (2010). Adaptive radiotherapy for bladder cancer reduces integral dose despite daily volumetric imaging. Radiother Oncol.

[12] Deutschmann H, Kametriser G, Steininger P (2012). First clinical release of an online, adaptive, aperture-based image-guided radiotherapy strategy in intensity-modulated radiotherapy to correct for inter- and intrafractional rotations of the prostate. Int J Radiat Oncol Biol Phys.

[13] Kensen CM, Janssen TM, Betgen A (2022). Effect of intrafraction adaptation on PTV margins for MRI guided online adaptive radiotherapy for rectal cancer. Radiat Oncol.

[14] Åström LM, Behrens CP, Storm KS, Sibolt P, Serup-Hansen E (2022). Online adaptive radiotherapy of anal cancer: Normal tissue sparing, target propagation methods, and first clinical experience. Radiother Oncol.

[15] Foroudi F, Wong J, Kron T (2011). Online adaptive radiotherapy for muscle-invasive bladder cancer: Results of a pilot study. Int J Radiat Oncol Biol Phys.

[16] Christiansen RL, Dysager L, Hansen CR (2022). Online adaptive radiotherapy potentially reduces toxicity for high-risk prostate cancer treatment. Radiother Oncol.

[17] Ray X, Moazzezi M, Bojechko C, Moore KL (2020). Data-driven margin determination for online adaptive radiotherapy using batch automated planning. Int J Radiat Oncol Biol Phys.

[18] de Jong R, Crama KF, Visser J (2020). Online adaptive radiotherapy compared to plan selection for rectal cancer: Quantifying the benefit. Radiat Oncol.

[19] Byrne M, Archibald-Heeren B, Hu Y (2022). Varian ethos online adaptive radiotherapy for prostate cancer: Early results of contouring accuracy, treatment plan quality, and treatment time. J Appl Clin Med Phys.

[20] Zwart LGM, Ong F, Ten Asbroek LA (2022). Cone-beam computed tomography-guided online adaptive radiotherapy is feasible for prostate cancer patients. Phys Imaging Radiat Oncol.

[21] Archambault Y, Boylan C, Bullock D (2020). Making on-line adaptive radiotherapy possible using artificial intelligence and machine learning for efficient daily re-planning. Med Phys Int J.

[22] Sheng Y, Li T, Lee WR, Yin F-F, Wu QJ (2017). Exploring the margin recipe for online adaptive radiation therapy for intermediate-risk prostate cancer: An intrafractional seminal vesicles motion analysis. Int J Radiat Oncol Biol Phys.

[23] Morgan HE, Wang K, Yan Y (2022). Reducing PTV margins with daily adaptive radiotherapy to the prostatic fossa. Int J Radiat Oncol Biol Phys.

[24] de Muinck Keizer DM, Kerkmeijer LGW, Willigenburg T (2020). Prostate intrafraction motion during the preparation and delivery of MR-guided radiotherapy sessions on a 1.5T MR-Linac. Radiother Oncol.

[25] Morgan HE, Wang K, Yan Y (2023). Preliminary evaluation of PTV margins for online adaptive radiation therapy of the prostatic fossa. Pract Radiat Oncol.

[26] Langen KM, Willoughby TR, Meeks SL (2008). Observations on real-time prostate gland motion using electromagnetic tracking. Int J Radiat Oncol Biol Phys.

[27] Lovelock DM, Messineo AP, Cox BW, Kollmeier MA, Zelefsky MJ (2015). Continuous monitoring and intrafraction target position correction during treatment improves target coverage for patients undergoing SBRT prostate therapy. Int J Radiat Oncol Biol Phys.

[28] Steiner E, Georg D, Goldner G, Stock M (2013). Prostate and patient intrafraction motion: Impact on treatment time-dependent planning margins for patients with endorectal balloon. Int J Radiat Oncol Biol Phys.

[29] Varian Medical Systems (2019) Ethos Algorithms Reference Guide

[30] Li HS, Chetty IJ, Enke CA (2008). Dosimetric consequences of intrafraction prostate motion. Int J Radiat Oncol Biol Phys.

[31] Adamson J, Wu Q (2009). Inferences about prostate intrafraction motion from pre- and post-treatment volumetric imaging. Int J Radiat Oncol Biol Phys.

[32] Boda-Heggemann J, Köhler FM, Wertz H (2008). Intrafraction motion of the prostate during an IMRT session: A fiducial-based 3D measurement with cone-beam CT. Radiat Oncol.

[33] Crehange G, Mirjolet C, Gauthier M (2012). Clinical impact of margin reduction on late toxicity and short-term biochemical control for patients treated with daily on-line image guided IMRT for prostate cancer. Radiother Oncol.

[34] Brand VJ, Milder MTW, Christianen MEMC, Hoogeman MS, Incrocci L (2022). Seminal vesicle inter- and intra-fraction motion during radiotherapy for prostate cancer: A review. Radiother Oncol.

[35] Kron T, Thomas J, Fox C (2010). Intra-fraction prostate displacement in radiotherapy estimated from pre- and post-treatment imaging of patients with implanted fiducial markers. Radiother Oncol.

[36] Smith GA, Dunlop A, Barnes H (2022). Bladder filling in patients undergoing prostate radiotherapy on a MR-linac: The dosimetric impact. Tech Innov Patient Support Radiat Oncol.

[37] Gardner SJ, Wen N, Kim J (2015). Contouring variability of human- and deformable-generated contours in radiotherapy for prostate cancer. Phys Med Biol.

[38] Engels B, Soete G, Gevaert T, Storme G, Michielsen D, De Ridder M (2014). Impact of planning target volume margins and rectal distention on biochemical failure in image-guided radiotherapy of prostate cancer. Radiother Oncol.

[39] Engels B, Soete G, Verellen D, Storme G (2009). Conformal arc radiotherapy for prostate cancer: Increased biochemical failure in patients with distended rectum on the planning computed tomogram despite image guidance by implanted markers. Int J Radiat Oncol Biol Phys.

[40] Hu Y, Byrne M, Archibald-Heeren B, Collett N, Liu G, Aland T (2020). Validation of the preconfigured Varian Ethos Acuros XB Beam Model for treatment planning dose calculations: A dosimetric study. J Appl Clin Med Phys.

[41] Netherton T, Li Y, Gao S (2019). Experience in commissioning the halcyon linac. Med Phys.

